# The characteristics, influence factors, and regulatory strategies of growth retardation in ruminants: a review

**DOI:** 10.3389/fvets.2025.1566427

**Published:** 2025-03-26

**Authors:** Tao Li, Bakhtawar Riaz Raja, Jie Liao, Longqing Zheng, Fuquan Yin, Shangquan Gan, Xuemei Sun, Gang Lyu, Jian Ma

**Affiliations:** ^1^College of Coastal Agricultural Sciences, Guangdong Ocean University, Zhanjiang, China; ^2^Xinjiang Taikun Group Co., Ltd., Changji, China

**Keywords:** ruminant, growth retardation, nutritional regulation, oxidative stress, gastrointestinal function

## Abstract

Growth retardation represents a main barrier to affect the productivity and efficiency of ruminants production, which is characterized by low growth rate, a disparity between skeletal and physiological maturation, gastrointestinal dysfunction and reduced reproductive performance. This review provides a concise overview of growth retardation in ruminants, and summarizes the key factors that influence their growth and development, including genetics, nutrition, microbiota and environment. Also, this review emphasizes the central role of nutritional management and gastrointestinal development, as well as the regulatory mechanisms involved in growth processes. In addition, recent advances in these aspects are discussed to form an integrative framework aimed at improving physiological function in ruminants. This review provides a comprehensive perspective for understanding the complex mechanism of growth retardation in ruminants, puts forward a theoretical basis for optimizing the production efficiency of ruminants industry and emphasizes the importance of multidisciplinary collaboration to provide a reference for advancing systematic research on growth and development of ruminants.

## 1 Introduction

Growth retardation has been a critical issue in livestock production, which affects animal welfare, reduces productivity and economic benefits and restricts the development of ruminants industry (1). A common strategy to enhance growth performance of animals is to increase the energy content in the feed. Huo et al. (2) reported that cows fed high-energy diets exhibited approximately a 30% higher average daily weight gain compared to those fed low-energy diets. The interaction between genetics and nutrition has become a central focus of research as recent advances in molecular biology. Genetics and nutrition have improved our understanding of mechanisms involved in ruminants growth. A previous study has shown that the growth and development of ruminants were not only affected by genetic, nutritional and environmental factors, but also the genetic modification of nutrients was an effective strategy to regulate the growth and development of ruminants (3). The main causes of growth retardation in animals are nutritional deficiencies, particularly in protein, energy, vitamins, and minerals. These deficiencies highlight the need for nutritional interventions as they directly affect growth rate and body condition. Specific nutrients supplementation can improve growth performance and immune resistance in ruminants. However, the optimal nutritional programs can not completely mitigate growth retardation, because the healthy development of gastrointestinal tracts is the premise of normal growth and development of animals. The rumen, a unique digestive organ in ruminants, is critical for the digestion of high-fiber diets. Meanwhile, the gut plays a dual role in nutrients absorption and immune support (1). Although recent progress has been made in the regulation of growth in ruminants, most studies mainly focus on the effects of individual nutrients and feed formulations, and often these factors are analyzed in isolation from each other. The comprehensive analysis of the interaction in multiple regulatory measures remains scarce. Therefore, on the basis of published literature and our previous studies, this review primarily summarized recent findings in growth retardation of ruminants, concluded the influencing factors and mechanisms involved in the process of growth retardation and presented a systematic framework for alleviating the ruminants' growth retardation.

## 2 Comprehensive insights into growth and development of ruminants

**Figure 1:**
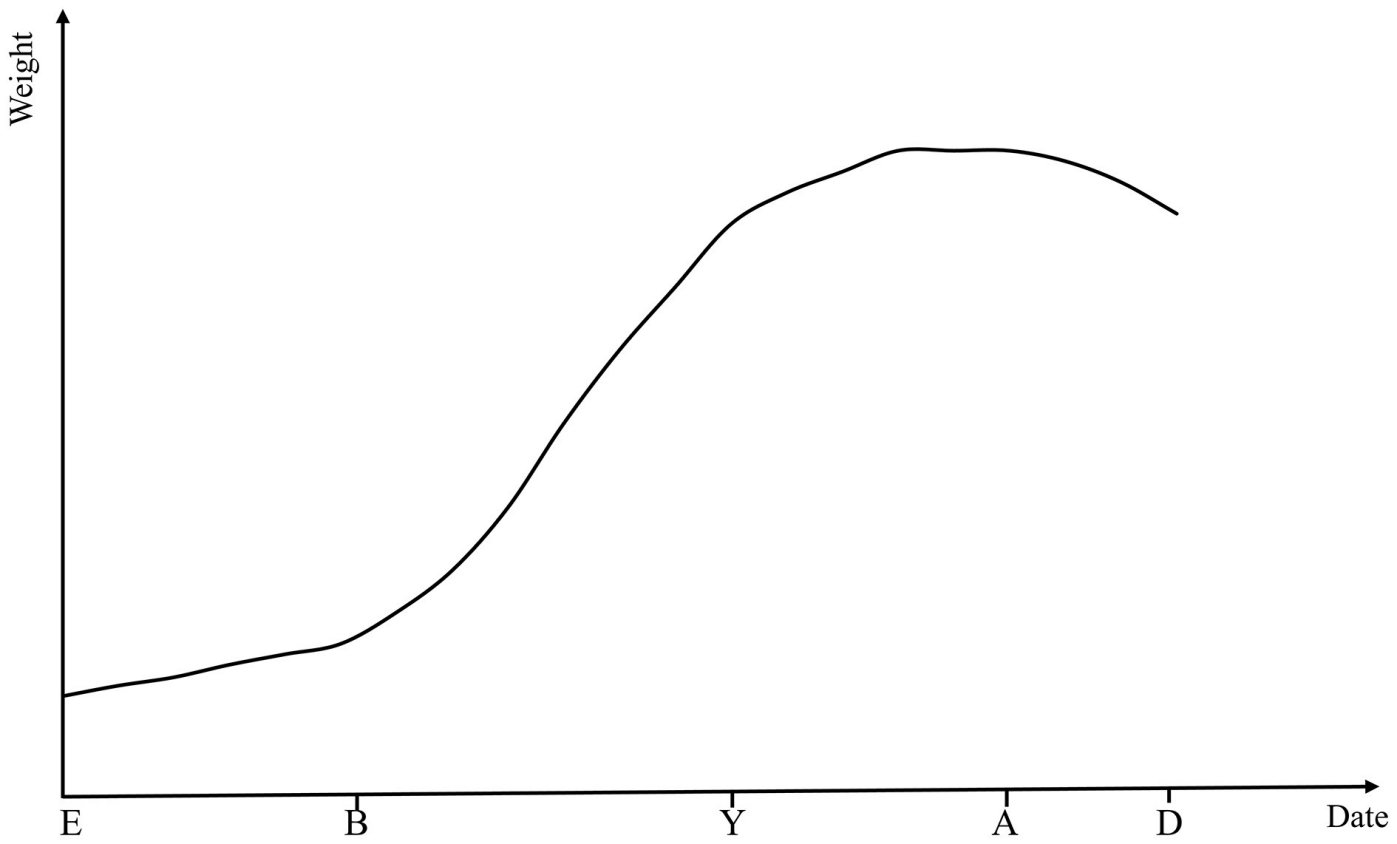
Theoretical growth rate curves across key stages of animal development. E, embryo; B, birth; Y, young; A, adult; D, death.

From the fertilized egg through embryonic, juvenile, and adult stages to senescence and death, individual growth and development is a lifelong process for animals. Throughout this life cycle, to support the ongoing development and functional optimization of tissues and organs, animals continuously accumulate nutrients and perform metabolic activities to maintain a dynamic balance in cell number and size. This process enables a continuous increase in body weight (BW) until the animal reaches its mature adult weight (4, 5). The growth and development of animals can be broadly divided into two phases including prenatal and postnatal stages. Individual growth status is commonly assessed through periodic measurements, which are conducted by observing age-related changes in overall weight, specific body parts, organs and tissues. The most obvious sign of growth is the continuous rise in BW with age, which is usually in the form of a distinctive “S”. Figure 1 shows a theoretical representation of growth rate curves over different time periods of animals. From birth to adolescence, animals grow rapidly and their body weight increases rapidly. From adolescence to adulthood, the growth rate of animals is slow, and their body weight remains stable in adulthood until death, when there is a slight decrease in body weight (6).

The interaction of genotype and environmental factors results in growth and development. Normal growth and development, with characteristic patterns of weight gain as well as unbalanced, non-isokinetic and sequential changes in body, are regulated by a balanced combination of nutrients, hormones, enzymes and a healthy physiological state (7). Genetic mutations, nutritional deficiencies, hormonal or enzymatic imbalances and drastic environmental changes often result in animals with BW or size that deviate from the population average for their breed and age. Unfortunately, the concept of growth retardation in ruminants remains undefined. In pigs research, Jones et al. (8) compared the physiological indexes between growth-retarded and normal pigs, and defined that pigs with BW in the lowest 10% were deemed to be growth retardation within the same breed and age. In human medicine, the World Health Organization classifies infants with a weight Z-score reduction >1.0 as growth retardation, defining it as a BW below the 5% normal infants or a growth rate two standard deviations below the norm (9, 10). In our previous studies of growth-retarded yaks, growth retardation referred to individuals whose growth performance is much lower than the average level under the condition of the same breed and age, which were mainly manifested as low BW, high mortality and reduced feed efficiency and immunity (11). Accordingly, in this review paper, in combination of relevant studies, growth retardation is defined as BW that the BW of one ruminant with the same breed and age is in the lowest 10% of population under the same environment and feeding conditions.

## 3 The characteristics of growth retardation in ruminants

### 3.1 Reduced weight gain and fat deposition ability

The weight gain rate is a key parameter to evaluate the production performance and health status of animals. Compared with normally developing animals, the growth-retarded ruminants typically have a lower BW, slower weight gain, poor muscle development, and limited fat deposition. Underweight mainly includes low birth weight and postnatal growth retardation due to genetic factor or nutrient deficiency, which results in a weight of <10% of the similar age (1). These characteristics have a direct impact on the final production efficiency and economic value of animals. As a central driver of glucose and lipid metabolism imbalances, adipose tissue dysfunction is closely linked to metabolic syndrome. Studies in growth-retarded animal models revealed that diminished fat deposition promotes pathogenic lipid redistribution, which exacerbates metabolic risks through mechanisms such as chronic inflammation (12). Nutritional deficiencies, including inadequate maternal nutrition or placental dysfunction leading to intrauterine growth retardation (IGR), are a major cause of low birth weight in ruminants. The IGR severely affects survival rate of ruminants after birth and can cause irreversible damage to subsequent production performance (13). Research of Zhang et al. (14) showed that placental dysfunction can reduce the efficiency of nutrients transport from the mother to the fetus. Consequently, IGR lambs had a significantly lower initial and final BW as well as reduced net weight gain and average daily gain when compared to lambs with normal intrauterine growth. Sutherland et al. (15) also reported that IGR had irreversible effects on normal growth trajectory, with IGR lambs having significantly lower BW after 127 days of birth. Early rearing ways, especially when the supply of essential nutrients for maintenance and growth is inadequate, are critical in determining later growth performance. Under such conditions, animals are unable to effectively synthesize the nutrients required for the formation of body tissues (16). In most animals, the weight at puberty is typically ~60% of the weight at maturity (7). A previous research by our group has demonstrated that the average daily gain and final BW of growth-retarded yaks were lower than those of normal yaks under same feeding condition (17). Inadequate management of ruminants causes slow weight gain and underdeveloped skeleton and muscles, especially thin subcutaneous fat layers on the back, abdomen and hip, which finally can lead to a long time to reach market weight.

### 3.2 Discrepancy in body size, bone age, and physiological maturity

The body size is an important index to assess the growth and development of animals, which indirectly reflects the developmental situation of organs and tissues. In ruminants production, the measurement of body size can be used for the selection of breeding stock and adjustment of feeding strategy. Normal ruminants have unbalanced and uneven growth characteristics. They generally grow from head to tail, then tail to head, and finally meet at the waist. In addition, until achievement of a coordinated balance, limb growth usually precedes trunk growth (14, 18). A mismatch between bone maturity and physiological age is often found in growth-retarded ruminants, which typically have shorter body length, smaller chest circumference and lower body height. Wang et al. (19) found that compared with normal yaks, the growth-retarded yaks had significantly lower body height, length, chest circumference and depth, as well as lower mean daily growth rate of body size. These reductions in skeletal dimensions are primarily attributed to growth hormone (GH) and insulin-like growth factor-1 (IGF-1) deficiencies, which impair chondrocyte proliferation in the growth plates of long bones (20, 21). A shorter body length is often correlated with delayed or poor skeletal development. A smaller chest circumference may indicate underdeveloped visceral organs (e.g., limited cardiopulmonary function), and a lower body height may indicate abnormal limb bone development (22). This imbalance has a negative impact on overall health, production performance, and economic benefit of ruminants. Research has shown that abnormal hormone secretion, malnutrition and genetic mutations, which collectively affect growth and development of animals, often contribute to the mismatch between bone maturity and physiological age (7).

### 3.3 Digestive system maldevelopment

Digestive tract health is influenced by macroscopic aspects of development and physiological integrity, as well as the microscopic balance between gut microbiota and immune status (23). Severe malnutrition in the early stage of life can damage the structure and function of the gastrointestinal tracts, leading to growth retardation in ruminants, which in turn limits the development of the digestive system (24). Study showed that growth-retarded yaks had a lower weight of rumen and intestine when compared to that of normal yaks (25). The rumen, a unique digestive organ of ruminants, has important function in digesting crude fiber. Ruminal microorganisms and enzymes produced by microorganisms can digest fiber into nutrients that can be utilized by the body, which has significant effects on growth of ruminants (26). In addition, the ruminal epithelium has a vital role in the absorption of volatile fatty acids (VFAs), and its healthy development is crucial for nitrogen transport and urea recycling (27). The height and width of rumen papillae are the important parameters to evaluate the relative development of ruminal epithelium. Our previous research found that compared with normal yaks, the growth-retarded yaks had a significantly lower ruminal papilla height and jejunal villus height, width, surface area and villus/crypt ratio (17). Moreover, another study in sheep has also shown that the small intestinal villus height and villus/crypt ratio of growth-retarded lambs were lower than those of normal lambs, suggesting that the gastrointestinal tracts of normal ruminants had a greater absorptive area and efficiency to absorb the nutrients, and then meet growth and production demands (28). Rumen dysgenesis can impair barrier function by reducing the expression of tight junction proteins (e.g., ZO-1 and Occludin) and increasing the risk of endotoxin and pathogens invasion (1, 23, 29). As a result, animals with growth retardation become more susceptible to microbial disturbances and inflammation, further hindering nutrient digestion and absorption.

Microbiota in the digestive system of animals are essential for growth, health and productivity and interact symbiotically with the host. Ruminal microbiota serve as the primary site for digestion and absorption of VFAs and excess ammonia nitrogen, while gut microbiota absorb nutrients that are not absorbed in the rumen and play a critical role in the immune system (30, 31). Underdeveloped digestive systems of ruminants is usually accompanied by a series of physiological function disorders, including imbalance of gastrointestinal microbiota. Then, some beneficial microorganisms can not be effectively established and maintained, leading to susceptibility to digestive diseases, reduced energy supply, impaired nutrient absorption, and ultimately inhibiting the growth performance of ruminants. Zhuang et al. (32) isolated a strain of *Bifidobacterium longum* (*B. longum* 1109) from calf feces using phenotype clustering analysis and strain isolation techniques, and mouse experiments demonstrated that this strain can promote the growth of young livestock by modulating microbial function and host metabolism, suggesting its potential application in animal nutrition. Du et al. (33) found that growth-retarded calves had a lower relative abundances of *Proteobacteria, Rhodospirillaceae, Campylobacter*, and *Butyricimonas*, which played an important role in energy and VFAs production, in the feces when compared to normal calves, whereas the pathogenic bacteria such as Anaeroplasma and Eubacterium were more abundant in growth-retarded calves. Our previous findings also showed that the relative abundances of bacteria involved in oligosaccharide, starch and cellulose degradation (e.g., *Ruminococcaceae, Spirochaetaceae, Clostridiaceae, Prevotellaceae*, and *Rikenellaceae*) were higher in normal yaks that those in growth-retarded yaks, while the *Christensenellaceae_R-7*, unclassified *Chitinophagaceae* and *Eubacterium* tenue were significantly higher in growth-retarded yaks (34). These results suggests that growth-retarded ruminants have a disrupted gastrointestinal microbiome, reduced gut immunity and nutrient utilization efficiency and increased susceptibility to digestive diseases such as diarrhea or enteritis, which further affects feed efficiency and growth rate.

### 3.4 Reduction of reproductive performance

The imbalance between body size and physiological development leads to a delayed sexual maturity and reduced reproductive performance. Many factors, including gene, nutritional status, environmental conditions and disease, can influence the reproductive performance of ruminants. Reproductive performance is regulated by the hypothalamic-pituitary-gonadal (HPG) axis, which controls gonadal function by regulating the secretion of gonadotropins, including the luteinizing hormone and follicle-stimulating hormone levels, thereby affecting follicular development, ovulation and spermatogenesis. The HPG axis is the core of the endocrine mechanisms that control the reproductive performance of ruminants and other mammals (35, 36). Growth-retarded animals often exhibit inadequate secretion of IGF-1 and GH, which affect reproductive performance together with the HPG axis. A previous study has shown that IGF-1 has a critical role in mammary gland development and milk synthesis, particularly in the regulation of fat and protein content (37). Ruminants with growth retardation have abnormal BW and reduced milk quality, which significantly affect offspring survival rates, which indirectly leads to a reduction in reproductive performance. In addition, *in vivo* studies have shown that IGF-1 and GH can directly act on the regulation and growth of bovine follicles at different stages, thus promoting follicular development (38, 39). Hormonal imbalances severely affect overall reproductive efficiency, leading to delayed sexual maturity, irregular estrous cycles, reduced embryo survival after conception and increased risk of abortion. The GH also regulates the development and metabolism of the male gonads through growth-promoting effects. Males with growth retardation tend to have smaller body size and underdeveloped gonads compared to normal females, which result in mating difficulty. IGF-1 protects sperm cells from oxidative stress through the antioxidant properties (40, 41). Kumar et al. (42) found that IGF-1 can increase the stability of the structure and function of buffalo sperm after cryopreservation, which significantly improved the fertilization ability of the sperm. This finding suggested that IGF-1 increased the activity of antioxidant enzymes in sperm cells, reduced the accumulation of free radicals, improved the viability of sperm and enhanced the reproductive performance of the male. In conclusion, the decline in reproductive rates in growth-retarded ruminants is primarily due to hormonal imbalances involving IGF-1, GH and the HPG axis, resulting in poor development of oocyte, sperm and gonad.

**Figure 2:**
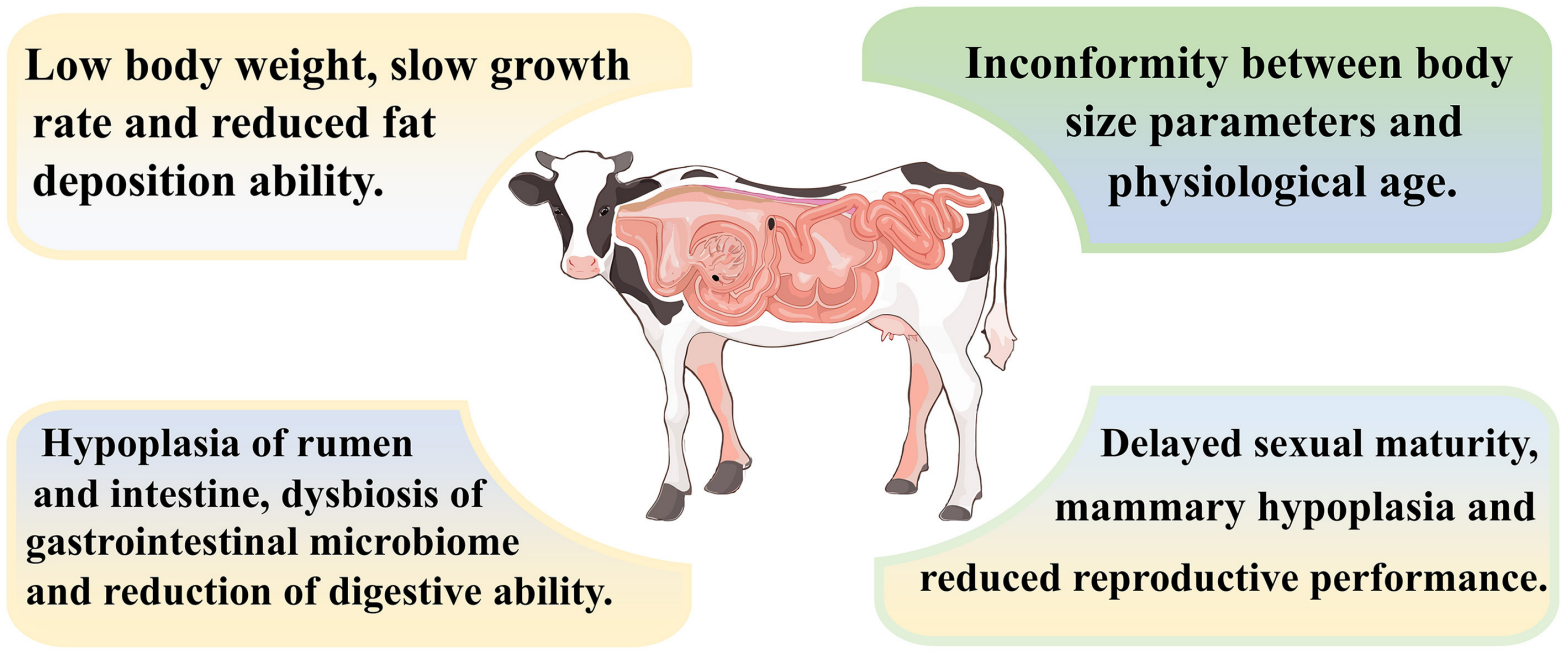
Characteristics of growth-retarded ruminants.

Overall, as shown in Figure 2, ruminants with growth retardation exhibit characteristics such as low body weight, slow growth rate and reduced fat deposition ability. In addition, the rumen and intestine of growth-retarded ruminants are dysplastic, and the nutrients digestion and absorption are decreased.

## 4 Key factors contributing to growth retardation in ruminants

### 4.1 Genetic determinants of growth in ruminants

Ultimately, gene expression and regulatory changes control the growth and development as well as metabolic processes (43). At the molecular level, a large number of genes and their associated signaling pathways play a critical role in cell proliferation, differentiation, nutrient metabolism and endocrine regulation during the growth of animals. The growth and development of animals will be significantly affected by abnormal mutations or expression of certain genes. Hu et al. (25) analyzed the effects of growth hormone-releasing hormone (GHRH) in the pituitary gland and the levels of GH and insulin-like IGF-1 in the blood between growth-retarded and normal yaks, and found a significant correlation between IGF-1 concentration and BW gain. Similarly, study by Saleh et al. (44) showed that the insulin-like hormone IGF-1 helped cells grow and differentiate, and it was also a regulator of cell development and DNA synthesis. GH is secreted by the pituitary gland and stimulates the liver to produce IGF-1. Then, IGF-1 binds to its receptor IGF1R, which activates the downstream PI3K/Akt and MAPK signaling pathways to promote cell proliferation and growth (45, 46). Li et al. (47) has shown that the growth of animals muscle fiber occurred primarily through protein deposition, which promoted muscle fiber hypertrophy, and IGF-1 stimulated protein synthesis via the PI3K/Akt/mTOR signaling pathway to increase muscle fiber cross-sectional area and promote muscle hypertrophy, while IGF-1 level decline inhibited this process. Meanwhile, Hu et al. (25) showed that injection of GH releasing peptide-2 (GHRP-2) into growth-retarded yaks significantly increased serum levels of GH and IGF-1, which not only improved the muscle fiber morphology of the longissimus dorsi and semitendinosus muscles, but also upregulated the expressions of PI3K, Akt, and mTOR mRNA, finally contributing to improved growth of growth-retarded yaks. Another experiment has also verified that long-term utilization of GHRP-2 significantly increased daily weight gain in Holstein cows, with increase of up to 36.4% (48). GHRP-2 is a GH secretinotropin peptide, and GH is the natural ligand of GHRP, which can act on internal receptors and stimulate the secretion of GH and IGF-1 (49). On the other hand, IGF-1 promotes muscle protein synthesis by activating the PI3K/Akt/mTOR pathways and increasing the phosphorylation of 4E binding protein (4EBP-1) and S6 kinase (S6K1) (50). Therefore, the abnormal secretion of GH and IGF-1 hormones is an important factor to induce growth retardation in ruminants, which not only causes slow weight growth and body size, but also indirectly results in the reproductive performance to be reduced.

In addition to the significant effects of GH and IGF-1 on cell proliferation and protein synthesis, other genes play a critical role in the regulation of ruminal development and skeletal growth, thereby affecting the growth of animals. Meng et al. (51) study found that gene knockout of the G-protein coupled receptor 41 (GPR41) in cattle significantly inhibited the ruminal epithelial cell proliferation and reduced the cyclin D2 and cyclin E2 levels. Ruminal cell proliferation has an important function in maintaining rumen homeostasis, enhancing rumen barrier and promoting nutrient digestion and absorption in ruminants. Additionally, myostatin (MSTN), a negative regulator of muscle growth, has been associated with skeletal size variation. Its inactivation significantly alters the gut microbiota composition in sheep, enhancing growth without compromising meat quality (52). Liu et al. (53) analyzed Notch1 gene polymorphism in Qinchuan cattle and found that five novel SNP loci (g.A48250G, g.A49068G, g.A49239C, g.C49307T, g.C49343A) were significantly associated with growth traits including body height, weight and hip height, which suggested that Notch1 can be used as a candidate gene for beef cattle breeding program. Kong et al. (54) investigated the regulatory role of long non-coding RNAs (lncRNAs) in the liver of Leiqiong cattle and identified differential expression of 1,124 mRNAs and 24 lncRNAs between growth-retarded and normal Leiqiong cattle. The differentially expressed mRNAs were not only associated with growth factor binding, K63-linked ubiquitination, and cellular protein metabolism, but also significantly correlated with growth and development pathways such as the PPAR signaling pathway and vitamin B6, glyoxylate and dicarboxylate metabolism. Among the lncRNAs, Lnc_002583 was found to play a synergistic regulatory role in the growth and development of Leiqiong cattle by influencing the expression of IFI44 and IFI44L genes. Yang et al. (55) identified CircDYRK1A in the muscle tissue of beef cattle and demonstrated its role in muscle development. Further analysis revealed that the CircDYRK1A-miR-21-5p-KLF5 axis regulates myoblast differentiation, providing a theoretical basis for genetic improvement in beef cattle breeding. In a recent study, genome-wide association analysis and quantitative trait loci mapping in sheep were used to identify MAP3K1, ANKRD55, ABCB1, MEF2C, and TRNAW-CCA-8 as key regulators of growth and development. These five candidate genes were significantly associated with BW traits in sheep, offering valuable insights into the genetic mechanisms underlying BW variation and their potential applications in selective breeding (56). In addition, studies have reported that fibroblast growth factors (FGFs) are critical in regulating bone and cartilage development in animals. Mutations in FGFs can cause cartilage developmental disorder of animals, resulting in skeletal maldevelopment which were manifested as dwarfism and growth retardation (57, 58).

To sum up, genetic factors significantly influence the healthy growth of ruminants. The interplay of hormones and genes, together with environmental and nutritional factors, regulate gene expression through epigenetic mechanisms, ultimately affecting individual growth and development.

### 4.2 Nutritional influences on growth and development of ruminants

#### 4.2.1 Disparity between nutrient supply and growth demand in ruminants

Nutrient imbalance refers to a situation where the nutrients consumed by animals do not meet their actual physiological requirements during the rearing process. This imbalance has a direct impact on health and production performance of animals. During the growth and development of ruminants, the barrier of nutrient transfer between dam and offspring, as well as interaction between different nutrient components in the diet, are major contributors to the mismatch of nutrient supply and demand.

##### 4.2.1.1 Barrier of nutrient transfer from dam to offspring

Pregnancy and lactation in dams are critical for the growth and development of their offspring. During pregnancy, placental dysfunction is a major cause of fetal malnutrition, which not only reduces survival rate of offspring, but also affects the economic benefits of livestock production (59). A previous research has shown that placental dysfunction can slow blood flow in the uterus and umbilical cord, impair the transport of oxygen and amino acids across the placenta, and reduce the concentration of growth factors involved in metabolic processes in the fetal circulation, ultimately limiting the fetal growth and development. This dysfunction is a primary factor responsible for fetal growth deviating from genetic trajectories and preventing the achievement of genetically determined growth potential (60, 61).

In sheep, trophoblast cells invasion and differentiation during embryo implantation are critical for placentation and fetal development. Study has shown that the chemokine CXCL12 regulated the placental implantation and growth through its receptor CXCR4, and inhibition of the CXCL12/CXCR4 pathway increased the genes expression of fetal FGF2, vascular endothelial growth factor (VEGF-A) and placental growth factor (PLGF), which subsequently tended to increase the placental weight and the number of placental clusters (62). The increase in the weight and number of placentas can increase the flow of blood through the umbilical cord and enhance the expression of placental glucose and amino acid transporters, which is conducive to improving the delivery of maternal oxygen and nutrients to the fetus (63, 64). Moreover, Britt et al. (65) fed pregnant ewes with tall fescue seeds infected by endophytes and analyzed placental gene expression, and results showed that endophyte infection altered the expression of microRNA-21 in the placenta. MicroRNA-21 is a potential biomarker of fetal hypoxia and its up-regulated expression is associated with placental pathology, including placental insufficiency and fetal growth restriction. During fetal development, skeletal muscle development is highly sensitive to nutrients supply. Inadequate nutrients supply will reduce the number of skeletal muscle fibers and alter fiber composition, which then affect fetal fat deposition and production performance (66, 67). This is because that the muscle fibers of animals are derived from myoblasts proliferation and differentiation in early fetal development. A decrease in the effectiveness of nutrients and growth factors impairs the periodic activity of myoblasts, especially inhibition in the function of leucine transporters that uniquely regulate muscle protein synthesis, thereby reducing the number of nucleus of each muscle fiber, resulting in fewer muscle fibers in the offspring, and ultimately affecting normal growth and development (68–72).

The genetic background of animals determines its specific nutrient requirements, metabolic pathways and absorption efficiency. Nutrition, in turn, can regulate gene expression, thereby influencing growth and reproductive performance of animals. The interaction between genetics and nutrition is essential to support growth and development of animals (73). Li et al. (74) highlighted that maternal supplementation with fish oil during late pregnancy in mammals can enhance the expression of the GPR120 signaling pathway, improve placental nutrient transport efficiency and promote the growth and development of offspring. In addition, studies have shown that fat deposition in animals was also regulated by the Wnt signaling pathway. During muscle formation in ruminants, Wnt signaling will be enhanced and the activity of PPARγ is regulated by the Wnt pathway via GSK-3β and β-catenin. When maternal nutrient supply is inadequate or placental function is compromised, GSK-3β activity will be reduced to cause proteolysis of β-catenin and suppression of PPARγ target gene expression, which affects muscle growth and fat deposition, resulting in lower birth weight and intrauterine growth retardation (75–77).

The growth and development of the offspring are directly influenced by the quality and quantity of the maternal milk. Milk is not only rich in proteins, fats, minerals, and other nutrients that provide the energy and necessary nutrients for the growth and development of neonate, but also contains several immune factors, such as immunoglobulins, lactoferrin and lysozymes, that boost the newborn immunity and protect it from disease (78). Among the polyunsaturated fatty acids, arachidonic acid (AA), eicosapentaenoic acid (EPA), docosapentaenoic acid (DPA), and docosahexaenoic acid (DHA) are the highest concentrations in colostrum, which is attributed to that AA and DHA are components of neurophospholipid membranes and critical for the development of the central nervous system and vision (79). Therefore, the high content of unsaturated fatty acids in colostrum is essential for the health, growth and development of ruminants in the later stages. Jagusch and Mitchell (80) studied the effect of lambs on the utilization of metabolic energy from maternal milk by using a comparative slaughter method, and results showed that lambs fed the lowest level of nutrition mobilized body fat and had a negative energy balance, besides, the rate of weight gain of these lambs was lower than that of lambs in a positive energy balance. After weaning, the nutrients that satisfy the growth and development of ruminants mainly come from feed. Carbohydrates in the feed are fermented in the rumen to produce VFAs, which provide energy for the body. Proteins are decomposed into amino acids and ammonia by microorganisms to provide raw materials for synthetic proteins and microbial proteins. Trace elements and minerals play an important role in the regulation of GHs and enzymes. In the cattle research of Sakase et al. (81) feeding a high-energy diet to calves after birth increased the concentration of insulin-like peptide 3 (INSL3), which in turn increased IGF-1 levels and promoted growth and muscle development.

Although numerous nutrition studies suggested that high-energy diets can regulate physiological status, promote growth and development, increase meat and milk production and improve meat quality (82, 83). Ford et al. (84) claimed that in growth-retarded lambs, there was still an imbalance between muscle and fat deposition, even under a high energy diet condition, and besides, at 9 months of age, the ratio of muscle to fat of lambs was reduced by 25%, leading to maldevelopment of the body. This is because ruminants with growth retardation, due to early nutritional deficiencies, reorganize and redistribute the nutrients, resulting in restricted expression of relevant genes or pathways, which results in incomplete organ or tissue development, such as an underdeveloped rumen and small intestine, and reduces nutrient absorption efficiency (1).

##### 4.2.1.2 Effects of nutrient interactions on growth of ruminants

Nutrient balance, with different nutrients interacting synergistically or antagonistically, is critical to growth and development of animals. These interactions have a direct impact on the efficiency with which animals utilize the nutrients in the feed, ultimately affecting growth, health and productivity of animals. In ruminants, the interaction between energy and protein is particularly important. Insufficient fiber intake leads to a decrease in rumination and salivary secretion, which reduce the buffering capacity of the rumen and may induce ruminal acidosis. In addition, the change in ruminal pH can weaken the function of ruminal microorganisms, inhibit the activity of fiber-degrading bacteria and reduce feed digestibility (85). Consequently, insufficient energy intake causes the body's reserves of fat and protein to be metabolized into energy, which weakens their role in tissue construction and repair, suppressing growth and development of ruminants. As a key nutrient for maintaining health and production performance, protein is essential for growth, tissue repair and maintenance of physiological functions of animals (86). The digestion and absorption of protein occurs primarily in the form of amino acids and small peptides. Amino acids are classified as essential (EAA) or non-essential amino acids (NEAA) according to whether animals can synthesize them. The synergistic interactions between EAAs and NEAA are critical for maintaining protein synthesis and metabolic balance. In addition, interactions of other nutrients not only affect the bioavailability and metabolic pathways of the feed, but also play a key role in regulating overall health and productivity of animals. Table 1 shows the interactions between different amino acids and other nutrients.

**Table 1:** Interactions among amino acids and various nutrients.

**Items**	**Nutrients**	**Interacting nutrients**	**Function type physiological**	**Physiological function**	**References**
Amino acid	Lysine	Arginine	Antagonistic effect^a^	Affects protein synthesis and causes growth retardation and low immunity	(144, 145)
		Methionine	Synergistic effect	Improve growth rate and feed conversion rate	(146)
		Threonine	Synergistic effect	Participate in immunity and protein synthesis to support intestinal health	(147)
	Methionine	Cysteine	Synergistic effect	Jointly participate in the synthesis of glutathione and resist oxidative stress	(148)
	Glycine	Serine	Synergistic effect	Synthesis of precursors for other amino acids and biomolecules, involved in the synthesis of creatine, purine, and other important molecules	(1, 149, 150)
	Leucine	Isoleucine	Synergistic effect	Participate in the synthesis of muscle proteins and energy metabolism, balance three amino acids to promote muscle growth and prevent muscle breakdown	(151, 152)
		Valine			
Vitamin	Vitamin A	Zinc	Synergistic effect	Zinc can enhance the utilization of vitamin A and improve immunity	(153)
	Vitamin E	Selenium	Synergistic effect	Jointly participating in the antioxidant defense system can effectively reduce oxidative damage to cell membranes and prevent muscle diseases	(129)
Mineral	Calcium	Phosphorus	Synergistic effect	Support bone development and prevent osteoporosis	(154)
	Magnesium	Potassium	Synergistic effect	Participate in the regulation of neuromuscular function, muscle contraction, and maintenance of cardiac function	(154)
	Sulfur	Selenium	Synergistic effect	Jointly acting on antioxidant defense mechanisms, such as the synthesis of glutathione peroxidase, to combat oxidative stress	(154)
	Sodium	Chlorine	Synergistic effect	Jointly maintain fluid electrolyte balance, regulate blood pressure and acid-base balance, and ensure normal neuromuscular function	(154)
Fatty acid	n-3	n-6	Antagonistic effect^b^	Causing an increase in inflammatory response and affecting growth performance and health status	(155, 156)

^a^Lysine and arginine are antagonistic: Both are absorbed by the intestinal alkaline amino acid transport system (such as the CAT family proteins), and excess lysine competitively inhibits arginine absorption.

^b^The antagonistic effect of n-3 and n-6 fatty acids: Both are based on the conversion of Δ6 and Δ5 dehydrogenases to their respective active metabolites in the body, e.g. n-3 to EPA and DHA and n-6 to AA. The antagonistic effect of the two is mainly achieved by enzyme competition and metabolite confrontation.

#### 4.2.2 Impaired nutrients absorption and metabolism in ruminants

The VFAs produced by microbial fermentation in the rumen can be absorbed directly by the ruminal epithelial cells, providing ~80% of the energy requirements for ruminants (87). Additionally, the microbial proteins that are synthesized in the rumen can also reach the small intestine, where they are absorbed and utilized (88). If the rumen is underdeveloped, the colonization and function of the ruminal microbiota will be impaired, leading to difficulty in the digestion of complex carbohydrates such as cellulose or ruminal acidosis (34). Propionate, a fermentation product of the ruminal microbiota, can stimulate free fatty acid receptors in the colonic epithelial L-cells and promote the secretion of GLP-1. GLP-1, in turn, transmits signals to the hypothalamus that affect the appetite (89). Numerous studies have shown that the maturation of ruminal microorganisms, such as fiber (e.g., *Ruminococcus flavefaciens, Fibrobacter succinogenes*), starch (e.g., *Ruminobacter amylophilus, Streptococcus bovis*) and protein-degrading bacteria (e.g., *Prevotella ruminicola, Butyrivibrio fibrisolvens*), directly affect the digestibility of feed, thereby influencing the growth and health of ruminants (90–92). Furthermore, a previous study has shown that ruminal acidosis altered the permeability of ruminal epithelial cells and damaged the ruminal barrier, allowing endotoxins to enter the bloodstream, triggering systemic inflammation and reducing growth efficiency of ruminants (93). The ruminal epithelial cells play a vital role in regulating nutrients absorption and preventing harmful substances from crossing the epithelium into the bloodstream. Previously, a study suggested that the ruminal epithelial cells absorb VFAs via the monocarboxylate transporter (MCT1), which works with protons to facilitate transmembrane transport, thereby supporting cellular energy supply (94). Simultaneously, ruminal epithelial barrier function is maintained by tight junctions to ensure dynamic homeostasis between ruminal contents and cells and prevent the invasion of harmful pathogens. The changes in ruminal pH and nutritional status can damage the structural integrity of tight junctions, which lead to a decrease in the expression of ZO-1, occludin and claudin-1, thereby compromising epithelial barrier function (95). The dynamic balance between the external environment and the physiological metabolism of body is therefore closely linked and plays an essential role in maintaining health and growth of animals.

In ruminants, the normal microbiota in the intestine helps to metabolize large molecules from external and endogenous sources, such as rumen-protected proteins, microbial proteins, vitamins and minerals. A well-developed gut has tight cell junctions and a robust immune barrier that effectively prevents the invasion of pathogens and harmful substances (34, 96). However, if the gut is underdeveloped, these barriers can be compromised, increasing the possibility of bacterial, viral and parasitic infections and leading to gastrointestinal diseases such as enteritis and diarrhea (23). Previous research from our team compared the gastrointestinal microbiota of growth-retarded and normal yaks by using 16S rRNA gene sequencing, and principal coordinate analysis revealed significant differences in bacterial composition across all gastrointestinal segments. The normal yaks had a higher relative abundances of bacteria associated with oligosaccharide, starch and cellulose degradation when compared to the growth-retarded yaks (34). In addition, we observed that the villus height and crypt area in the intestine of growth-retarded yaks were significantly lower than those of normal yaks, while the expression levels of inflammatory factors such as tumor necrosis factor were significantly higher in growth-retarded yaks, indicating that the gastrointestinal barrier function was impaired in growth-retarded yaks (17). Balancing the gut microbiota and improving gut barrier function may be effective solutions to promote growth in growth-retarded ruminants. Previous studies found that the gut microbiota can promote the differentiation of immune cells through regulatory T cells and Th17 cells, and disruption of the gut microbiota can lead to bacterial translocation and impaired gut barrier function, which in turn affected overall health of animals (97, 98). Therefore, improving the ruminal and intestinal health are essential to increase nutrients utilization efficiency and promote growth in ruminants. In production, strategies such as optimizing feed formulations and using probiotics can help maintain microbial balance and gut health, thereby improving growth, development and health of ruminants.

### 4.3 Role of immunity and oxidative stress in health and growth of ruminants

Healthy problems, including chronic disease, parasitic infection, immune dysfunction and oxidative stress, induce the disturbance of energy balance and nutrient metabolism, ultimately affecting growth rate, weight gain, and mortality (99, 100). Chronic disease (e.g., rumenitis, enteritis, and pneumonia) trigger a sustained immune response that consumes significant energy reserves to maintain immune function, thereby reducing growth and production efficiency. In a study of growth-retarded lambs, the levels of inflammatory cytokines, including IL-1β, IL-6 and TNF-α, were significantly elevated in the blood when compared to normal lambs (101). In addition to the ability to inhibit the production of IGF-1 in the liver, these cytokines can activate the NF-κB pathway to suppress the hypothalamic-pituitary-gonadal-liver (HPGH) axis and GH secretion. IGF-1 is a key regulator of growth, and its deficiency directly limits the development of bones, muscles and organs in animals (102, 103). In addition, parasitic infection rapidly triggers an immune response that induces a Th2-type response and promotes the secretion of large amounts of pro-inflammatory cytokines (104). Razavi et al. (105) observed that coccidiosis infection in sheep induced an acute phase response and the liver to produce a large amount of acute phase proteins (APPs). This immune response leads to systemic metabolic reprogramming and causes the liver to increase glucose production through gluconogenesis to meet the energy needs of immune cells. However, the process results in weight loss and growth restriction. Meanwhile, toxins secreted by parasites such as protease inhibitors also inhibit the nutrients absorption and damage intestinal and liver cells, which further aggravate the metabolic burden of the host (104, 106).

Oxidative stress produces excessive reactive oxygen species (ROS) through lipid peroxidation, which can disrupt the integrity of lipid bilayer of cell membrane, alter enzyme activity, receptor function and conduction effects of signaling pathways. ROS can also attack mitochondria, cause mitochondrial autophagy via the Parkin and PTEN-induced kinase 1 (PINK1) pathways and impair cellular proteins and DNA (107–109). These effects will weaken mitochondrial dysfunction, affect ATP production and reduce energy supply, all of which limit growth and development of animals, particularly in tissues with high energy metabolism. Studies suggested that elevated IGF-1 levels activated the Nrf2/ARE pathway, which increased the expression of endogenous antioxidant enzymes such as GPx, helping to mitigate the physiological effects of ROS and alleviate oxidative stress (108, 110, 111). Therefore, IGF-1 not only promotes cell growth and differentiation, but also plays a key role in enhancing the antioxidant capacity and immune defense of the body, thereby indirectly supporting growth and development. In summary, effective management and disease prevention strategies aimed at improving oxidative stress resistance and immune function may significantly improve health and enhance growth and developmental efficiency of ruminants.

### 4.4 Environmental factors of growth in ruminants

Environmental factors are important external conditions that affect the growth and development of ruminants. These factors include stress (mental stress), temperature and humidity, light exposure, noise, air quality and stocking density, all of which can be categorized as environmental stressors. Sub-optimal environmental conditions can induce stress responses in animals that adversely affect physiological functions and metabolic processes, ultimately leading to growth retardation. In sheep, Du (112) found that maternal stress inhibited the development of fetal muscle and adipose tissue by enhancing DNA methylation within the promoter region of peroxisome proliferator-activated receptor-gamma coactivator 1-alpha (PGC-1alpha), and this epigenetic modification persisted in the muscle and fat tissues of the offspring and had long-lasting effects on their development. Bisphenol A (BPA) is a common environmental pollutant. Zhang et al. (113) found that pregnant ewes exposed by BPA reduced the placental and trophoblast progesterone (P4) levels, and besides, BPA exposure activated mitochondrial apoptotic pathways, which significantly reduced Bcl-2 levels and increased caspase-3 and−9 expression, ultimately reducing the number of viable cells in the placenta, inducing cell apoptosis and leading to IGR in lambs. Temperature also affects the reproductive performance of ruminants. A previous study reported that elevated temperatures can impair ovarian granulosa cell function in cows and cause oxidative damage and cell apoptosis, resulting in infertility and a negative impact on reproductive performance (114). Another factor that can affect fetal development is the ambient temperature during pregnancy. In hot environments, fetal umbilical blood flow will be reduced and the expression of the glucose transporter GLUT-8 in the placenta as well as insulin levels are significantly reduced, which limit fetal growth within the uterus (115, 116).

During the process of growth, animals become unable to effectively dissipate or generate heat when external temperatures exceeds their thermoregulatory capacity, which leads to a series of physiological and metabolic changes. In a high temperature environment, animals exhibit increased respiratory rates, reduced feed intake and elevated body temperatures, as well as increased tissue catabolism and reduced anabolism processes, which not only reduce nutrients intake, but also accelerate nutrients catabolism, ultimately leading to slow weight growth (117). In addition, heat stress disrupts the endocrine balance in animals by activating the hypothalamic-pituitary-adrenal (HPA) axis, which increases the secretion of corticotropin-releasing factor, adrenocorticotropic hormone and cortisol. Higher cortisol will increase blood glucose levels and catabolism and inhibit protein and fat synthesis, thereby reducing growth efficiency of animals (118, 119). Noordhuizen (120) reported that under heat stress conditions, each 1°C increase in temperature resulted in a 0.85 kg reduction in feed intake and a 600–900 kg reduction in annual milk production in dairy cows. Heat stress also causes a redistribution of internal resources, including fat, protein and energy, and alters absorption and metabolism regardless of changes in feed intake (121). Conversely, in a cold environment, the basal metabolic rate of animals will be increased and more energy is used to maintenance requirement, which reduce the efficiency of feed conversion and slow down growth rates. Our previous investigation found that the harsh plateau environment of the yaks during pregnancy and after birth, characterized by the depletion of forage resources, leads to inadequate early nutrition of the yak calves, which contributes significantly to growth retardation in later life (1). In addition, light exposure plays a key role in regulating growth, development, reproduction and behavior of animals. Photoperiod affects the secretion of melatonin, which in turn affects circadian rhythms and the secretion of GHs (122). Proper humidity levels, good ventilation and optimal stocking density are also crucial factors that contribute to the healthy growth and development of animals. Therefore, it is essential in livestock management practices to optimize environmental conditions, reduce stressors and provide the best conditions for growth and development.

According to the researches mentioned earlier, the growth and development of ruminants are affected by a variety of internal and external factors. These factors include changes in the structure and function of genetic materials, imbalances in nutrients supply and demand, interactions between genetics and nutrition, immune function, oxidative stress levels and environmental conditions, which are intricately interrelated and collectively influence hormone levels, endocrine regulation, nutrients digestion and absorption, energy metabolism, immune responses and oxidative stress, ultimately regulating the growth rate and health status of ruminants.

## 5 Regulation strategies of growth retardation in ruminants

### 5.1 Advancing growth performance and efficiency through genetic selection and improvement in ruminants

The important strategies for improving growth performance and disease resistance in ruminants are genetic selection and improvement. Genetic selection identifies and maintains individuals with superior growth traits and disease resistance, while genetic improvement utilizes modern molecular biology techniques to precisely modify the genome to achieve targeted improvements in specific traits. Gene editing technologies such as CRISPR-Cas9 have been widely used in the genetic improvement of livestock to improve growth rates, feed conversion efficiency, and disease resistance (123). The CRISPR-Cas9 system uses the Cas9 nuclease to recognize and cleave the target genome sequence using guide RNA to form DNA double-strand broken fragments, which rely on cells to initiate the connection or homologous recombination of non-homologous ends to achieve gene knockout or insertion (124). Recent successful animal gene editing applications have demonstrated that the ability to modify expression of genes such as IGF-1 and GH receptors can improve production efficiency and growth performance of animals (125, 126). In practical livestock production, hybrid vigor is also an effective approach to improving growth performance. Hybridization combines the beneficial traits of different breeds to produce offspring that are more adaptable and higher yielding. For example, the milk yield and growth performance of the hybrid cattle are significantly higher than that of the purebred individuals (127). The continued advancement and refinement of gene-editing technologies is therefore a powerful tool for improving growth and productivity of ruminants.

### 5.2 Optimizing feeding strategies and implementing targeted nutritional regulation for enhanced growth of ruminants

Proper nutritional management is essential to improve growth and production efficiency of ruminants. From a molecular nutrition perspective, nutrients regulate metabolic pathways and gene expression in ruminants, ultimately improving growth performance. Ruminants rely primarily on VFAs, produced by microbial fermentation of fibrous feedstuffs in the rumen, as the primary source of energy (87). A previous research found that the type of carbohydrate in the diet can influence the composition of the ruminal microbiota, thereby affecting the production and ratio of VFAs, which in turn modulates energy metabolism in the host (90). Our previous research also found that supplementing glutamine in the diet of growth retardation yaks can improve the function of the gastrointestinal barrier and promote compensatory growth in growth retardation yaks (11). EAA, NEAA, vitamins and minerals not only have different physiological functions, they also interact to produce synergistic effects that positively influence growth and development of animals. Supplementation of specific nutrients in the diet can alleviate growth retardation and improve growth performance in ruminants. Politis et al. (128) showed that adding 3,000 IU of vitamin E per cow per day to the diet of dairy cows effectively suppressed neutrophil and macrophage function in early postpartum, thus alleviating inflammation. Similarly, June et al. (129) found that supplementation of vitamin E and selenium significantly reduced blood malondialdehyde levels, increased superoxide dismutase activity, attenuated oxidative stress and improved feed intake in pregnant dairy heifers. These findings highlight the potential of appropriate nutritional supplementation to alleviate stress responses and regulate growth performance in ruminants.

Feed additives such as probiotics, prebiotics and synbiotics can also influence growth and development of ruminants. These additives regulate the composition and metabolic function of the gut microbiota to improve nutrients absorption and feed utilization efficiency. Some researches has confirmed that the addition of feed additives in diets can promote feed digestion, stabilize ruminal pH, strengthen the ruminal barrier and improve the gut microbiota, leading to improved feed intake and utilization (130–132). Zhang et al. (28) reported that the addition of L-arginine and N-carbamoyl-glutamine to the diet significantly increased the levels of ATP, ADP, total amino nitrogen (TAN) and AEC in the duodenum, jejunum and ileum of IGR lambs, and besides, the activity of key enzymes involved in the tricarboxylic acid (TCA) cycle was also enhanced, thereby improving gut function and the efficiency of feed digestion and absorption. Another study also showed that dietary supplemention with cysteine hydrochloride and active dry yeast reduced serum LPS levels, up-regulated the expression of genes related to VFAs absorption (SLC26A3, PAT1, MCT1) and cell junctions (CLDN1, CDH1, OCLN) in the rumen and down-regulated the expression of complement system genes (C2, C7) of growth-retarded yaks, which finally promoted growth (133). Sun et al. (134) revealed that rumen microbial metabolites, such as indole-3-carboxaldehyde and prostaglandin D2, regulate rumen development through specific signaling pathways, underscoring the pivotal role of microbial-metabolite interactions in precision nutrition. These findings lay a theoretical foundation for precision feeding strategies in young livestock by leveraging functional strain screening and targeted metabolite supplementation, providing new insights into the development of feed additives for young ruminants. In livestock production, the expression of growth-related genes can be effectively modulated by the rational regulation of dietary components through nutritional strategies to improve growth performance of ruminants. While the above studies highlight the positive effects of nutritional regulation on ruminants growth, they are often limited by small sample sizes and experimental conditions that may not fully reflect the complexity of different farming environments. Future research should include more comprehensive multi-factorial analysis to explore the synergistic effects of different nutrients or the interaction between nutrition and environment, with the aim of identifying optimal nutritional strategies.

### 5.3 Ensuring gastrointestinal microbial balance for promoting growth in ruminants

As a key factor in growth performance, the gastrointestinal microbiota plays a critical role in nutrient absorption, metabolism, and health of ruminants. Researches have shown that increasing fermentable fiber in the diet can stimulate the growth of fiber-degrading bacteria such as *Fibrobacter* and *Ruminococcus*, thereby promoting the production of acetic and butyric acids (91, 135). The diversity and functional composition of the gastrointestinal microbiota are closely related to feed digestibility, metabolic product formation and host gene expression (136). Our previous study found that the dietary addition of glutamine increased the relative abundance of unclassified *Peptostreptococcaceae, Romboutsia, Lachnospiraceae*, and *Clostridium* sensu stricto 1 in the gastrointestinal tract of growth-retarded yaks, and besides, the protease activity in the jejunum and ileum was also increased, which was beneficial for improving nutrients digestibility (137). Du et al. (33) reported that the weight gain, feed intake and GH/IGF-1 levels were significantly improved by adding probiotics in the diet of growth-retarded calves. Moreover, probiotics intervention increased the abundance of beneficial bacteria related to VFAs production, such as *Bacteroides, Prevotella, Campylobacter*, and *Butyrivibrio*, while reduced the prevalence of pathogens. Thus, regulating the composition of the gastrointestinal microbiota can improve nutrients digestibility, gastrointestinal fermentation, and digestive enzyme activity, thereby promoting growth and development of ruminants. In addition to contributing to nutrients metabolism, the gut microbiota also regulates host gene expression through metabolites such as VFAs and lactic acid. Studies have shown that VFAs, including acetic, propionic and butyric acids, can up-regulate the expression of tight junction proteins (occludin and claudin) in the intestinal epithelial cells via histone deacetylases (HDACs) and G protein-coupled receptors (GPR41 or GPR43), thereby enhancing intestinal barrier function. Activation of GPR41 also promotes the secretion of gut hormones such as GLP-1, which are involved in the regulation of energy balance and appetite in the host (89, 95, 138). Recent advances in microbiomics technologies have made it possible to precisely analyze how different nutritional strategies affect the composition and function of the microbiota, providing a scientific basis for optimizing feed formulations and improving production performance of animals (139).

### 5.4 Strategies for disease prevention and stress management in ruminants

In modern intensive farming systems, animals are often subjected to high density and production conditions. Under such circumstances, sub-optimal physiological conditions, including disease and stress, are major contributors to growth retardation and reduced production efficiency. Effective disease prevention and control measures that maintain the health status of ruminants can minimize production losses and improve growth performance and farm profitability. Razavi et al. (105) showed that when sheep were affected by diseases such as coccidiosis, the malondialdehyde and total homocysteine concentrations increased significantly, while the levels of superoxide dismutase and glutathione reductase were decreased, and the utilization of trace elements such as zinc, manganese and selenium was also reduced. Disease signals are transmitted to the hypothalamus, activating the hypothalamic-pituitary-adrenal axis and the sympathetic-adrenal axis, which triggers the release of glucocorticoids and catecholamines to stimulate macrophages and lymphocytes to produce large amounts of pro-inflammatory cytokines, thus causing an inflammatory response.

In addition, optimal environmental conditions and welfare standards not only help reduce stress responses and disease incidence, but also promote growth and development of animals. Ruminants have a limited range of tolerance to temperature and humidity, and unfavorable conditions can adversely affect their metabolism, immune system and growth performance. High temperatures can reduce feed intake, increase respiratory rates and induce heat stress, which negatively affects weight gain and milk production in dairy cows (118). Kaushik et al. (140) investigated the expression of heat shock protein 70 (HSP70) genes in goats of different ages, and found that HSP70 expression was highest at 9 months of age and decreased as the age increasing. Notably, HSP70 expression not only reflects the degree of heat stress, but is also closely associated with growth, energy metabolism efficiency and protein processing in animals (141, 142). Stresses, a common trigger for disease outbreaks and health problems, include transport, weaning, and feed transition. In practical farming, reducing environmental fluctuations, optimizing transport schedules, adjusting weaning procedures, and gradually changing diets can significantly reduce stress responses and the risk of disease outbreaks. Feed additives are widely used to reduce stress in animals. Su et al. (143) showed that renal cells from Mongolian sheep exposed to high-sugar conditions exhibited decreased vitality and increased levels of ROS. The addition of γ-aminobutyric acid (GABA) alleviated metabolic stress by enhancing glycolysis and promoting the TCA cycle, as well as up-regulating the expression of antioxidant genes to reduce oxidative damage in cells. Optimizing the rearing environment and improving animal welfare are essential strategies for promoting growth and improving farm efficiency of rumiannts. In highly modernized production systems, the timely integration of new technologies and equipment is essential to improve production efficiency and health of ruminants.

## 6 Future perspectives and concluding insights

**Figure 3:**
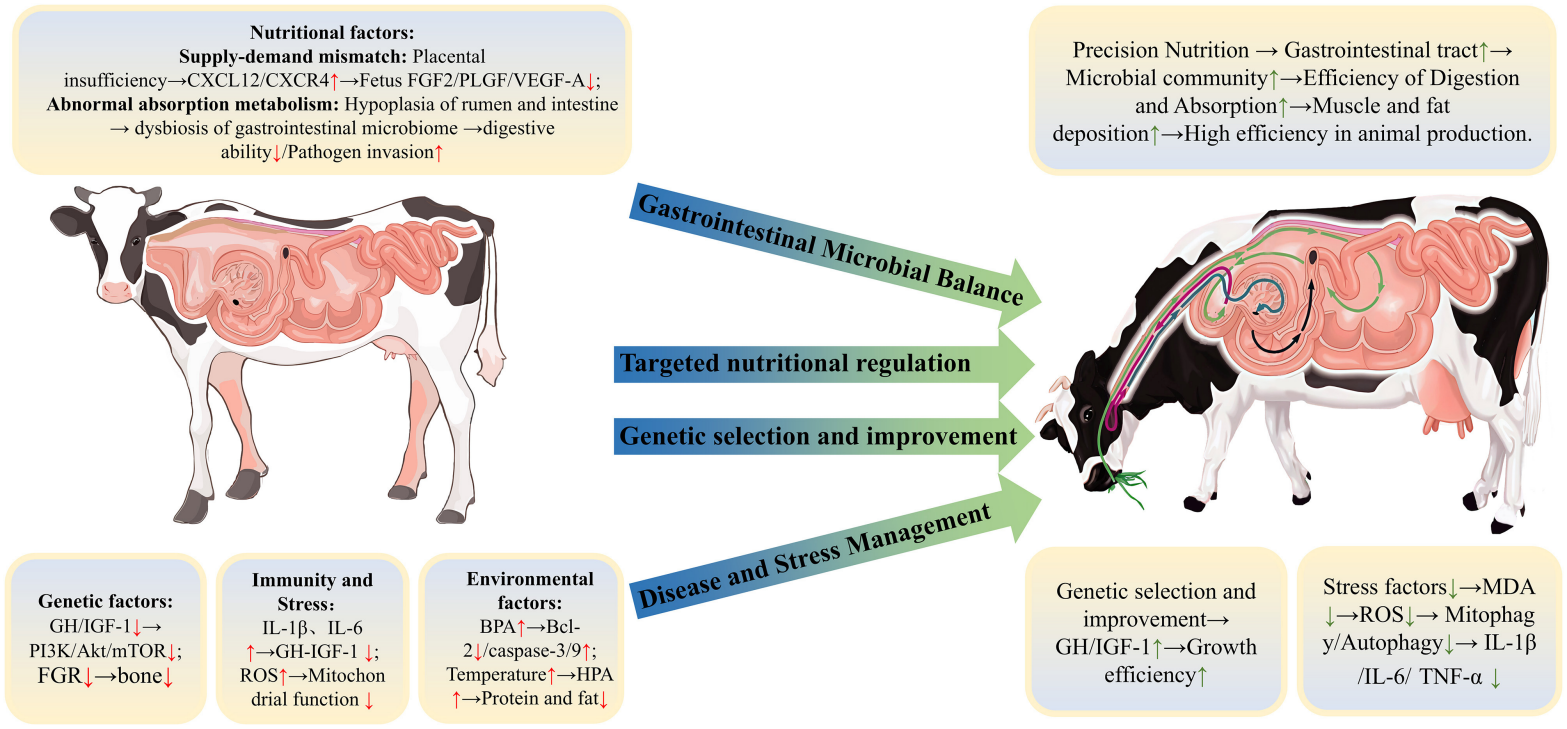
Factors affecting the growth and development of ruminants and control strategies.

Growth retardation poses a complex and multifaceted challenge in ruminants production, significantly impeding efforts to enhance productivity and efficiency. This review systematically defines and characterizes growth retardation, examines its extensive physiological impacts on ruminants, and analyzes the diverse factors influencing growth and development. As illustrated in Figure 3, the growth and development of ruminants are regulated by a complex interplay of factors, including genetics, nutrition, immunity, antioxidant capacity, and environmental conditions, which collectively shape the growth process. This review underscores the critical roles of nutrition and gastrointestinal health in fostering ruminants growth, integrating insights from genetics, nutrition, microbiology, and environmental sciences to reveal the synergistic effects of these factors on growth regulation. A holistic and multifactorial approach is therefore essential to optimize physiological functions and enhance production performance in ruminants. Additionally, fostering interdisciplinary collaboration that merges perspectives from genetics, microbiology, nutrition, and animal welfare is vital for building more efficient and sustainable livestock production systems. This synthesis highlights the importance of collaborative research that considers the interrelated nature of these factors, ultimately providing a basis for the development of targeted strategies to support sustainable growth.

Future research, guided by advances in genetic analysis, microbiome profiling and real-time health monitoring, should prioritize the development of precision-based approaches that tailor interventions to the specific needs of individual ruminants or herds. Emerging genomic and transcriptomic tools can further enable selective breeding for growth-enhancing traits, while novel nutritional strategies, such as targeted supplementation or prebiotic and probiotic interventions, can improve gut health and nutrient absorption. Continued investigation of the gut microbiome, particularly in relation to its interaction with immune function and stress resilience, offers promising avenues for improving animal health and growth performance of ruminants. Integrating stress management practices, such as optimized housing and handling systems, with nutritional and microbial interventions may contribute to a more holistic approach to mitigating growth retardation. Ultimately, the management of ruminants with growth retardation will benefit from a systems-level perspective that considers not only individual factors, but also how they collectively influence growth. The future of the field lies in advancing technologies and multidisciplinary collaborations that enable practical, evidence-based solutions and promote more productive, sustainable and humane ruminant production systems.
